# Physiological Processes Underlying Short Interval Intracortical Facilitation in the Human Motor Cortex

**DOI:** 10.3389/fnins.2018.00240

**Published:** 2018-04-11

**Authors:** Mehdi A. J. Van den Bos, Parvathi Menon, James Howells, Nimeshan Geevasinga, Matthew C. Kiernan, Steve Vucic

**Affiliations:** ^1^Westmead Clinical School, University of Sydney, Sydney, NSW, Australia; ^2^Brain and Mind Center, University of Sydney, Sydney, NSW, Australia

**Keywords:** short interval intracortical inhibition, short interval intracortical facilitation, TMS, CSP duration, ICF, circular coil, figure of eight coil, MEP amplitude

## Abstract

Short interval intracortical facilitation (SICF) may be elicited by a paired pulse transcranial magnetic stimulation (TMS) paradigm, whereby a suprathreshold first stimulus (S1) precedes a perithreshold second stimulus (S2). Other facilitatory circuits can be probed by TMS such as intracranial facilitation, however the cortical contributions to these circuits may lie partially outside of M1. SICF as such represents a unique analog to M1 inhibitory circuits such as short interveal intracortical circuits. The aim of the present study was to provide insight into the physiological processes underlying the development of SICF using the threshold tracking TMS technique which was recently demonstrated to have significant reliability. TMS studies were undertaken on 35 healthy controls, using either a 90 mm circular and 70 mm figure of eight coil, and one of two targets (0.2 and 1.0 mV) tracked. The motor evoked potential (MEP) responses were recorded from the abductor pollicis brevis. SICF was consistently evident between interstimulus intervals (ISI) of 1–5 ms (*P* < 0.001), with two peaks occurring ISIs 1.5 and 3 ms when using the circular coil. A significant SICF reduction (*F* = 5.631, *P* < 0.05) was evident with the higher tracking target, while SICF increased when stimulating with the figure of eight coil. While there was a correlation between SICF and CSP duration, there was no relationship between SICF and SICI or ICF. Age appeared to have no influence on SICF, SICI, or ICF. Findings from the present work suggest that SICF appears to be mediated by I-wave facilitation.

## Introduction

Cortical excitability reflects a balance between inhibitory and facilitatory neuronal circuits projected through pyramidal tract output tracts (Di Lazzaro et al., 2008; Ni and Chen, 2008; Rusu et al., 2014). Transcranial magnetic simulation (TMS) applied to the primary motor cortex (M1) has become widely utilized to assess cortical physiology in healthy controls and human disease (Rossini et al., 1999, 2015; Chen et al., 2008; Vucic et al., 2013). Importantly, the TMS technique can assess different inhibitory and facilitatory circuits within M1 using paired-pulse paradigms (Vucic et al., 2013; Rossini et al., 2015; Ziemann et al., 2015). When a subthreshold conditioning stimulus (CS or S1) precedes a suprathreshold test stimulus (S2), at interstimulus intervals (ISI) of 1–7 ms, the motor evoked potential (MEP) produced by the test stimulus becomes inhibited, termed short interval intracortical inhibition (SICI; Kujirai et al., 1993; Vucic et al., 2006). Increasing the ISI from 8 to 30 ms leads to facilitation of the MEP response, termed intracortical facilitation (ICF; Kujirai et al., 1993; Vucic et al., 2006).

In contrast, increasing the S1 intensity toward peri- and suprathreshold levels, followed by an S2 stimulus at perithreshold intensity, leads to synergistic levels of facilitation, a phenomenon termed short interval intracortical facilitation (SICF; Tokimura et al., 1996; Ziemann et al., 1996c). SICF develops over short ISIs (1–5 ms) with three distinct peaks at ISI 1–1.5, 2.4–2.9, and >4.5 ms (Tokimura et al., 1996; Ziemann et al., 1996c; Chen and Garg, 2000). While the physiological processes underlying SICF remain to be clarified, a cortical origin has been proposed (Ziemann et al., 1998a). Specifically, TMS stimulation results in descending corticospinal volleys, which are composed of direct (D) and indirect (I) waves (Day et al., 1989; Burke et al., 1993; Di Lazzaro et al., 1998a,b, 2003, 2008, 2012; Hanajima et al., 2002; Rusu et al., 2014; Rossini et al., 2015). Modeling studies have suggested that I-waves are produced through trans-synaptic interaction between cortical interneuronal circuits and pyramidal cells (Rusu et al., 2014). Facilitatory I-wave interactions produced by S1 and S2 stimulation within the motor cortex have been postulated as a likely physiological mechanism for SICF, a notion supported by epidural recordings (Di Lazzaro et al., 1999). Of further relevance, pharmacological studies have established a reduction of SICF by GABA and dopamine agonists, suggesting the importance of GABAergic and dopaminergic circuits in SICF generation (Ziemann et al., 2015). Separately, it has also been reported that disinhibition of neuronal circuits mediating I-wave generation could contribute to SICF development (Wagle-Shukla et al., 2009).

Of further relevance, an increase in the test stimulus intensity was reported to increase SICI and reduce SICF and ICF (Sanger et al., 2001; Daskalakis et al., 2002; Wagle-Shukla et al., 2009). It was postulated that these effects were mediated at a cortical level, with SICI enhancement resulting from inhibition of late I-waves recruited at higher intensities (Di Lazzaro et al., 1998b). The ICF reduction appears related to activation of distinct cortical neurons less sensitive to facilitation or located distant from the stimulation site (Volz et al., 2015). In addition, the reduction of SICF with increasing TMS intensity was ascribed to depletion of the subliminally depolarized cortical neuronal pool (Wagle-Shukla et al., 2009), important for SICF generation. In contrast, higher conditioning (S1) intensities in some SICI protocols have also been associated with activation of SICF and a reduction of SICI (Peurala et al., 2008), suggesting a potentially “contaminating” effect of SICF on inhibition. Separately, an influence of later SICI segments on ICF has also been reported at higher stimulus intensities (Hanajima et al., 1998).

A potential reason for discordant findings may relate to use of the constant stimulus method in assessing intracortical inhibition and facilitation. Recently threshold tracking TMS has been shown to be more reliable than the constant stimulus method (Samusyte et al., 2018). To better consider the processes that underlie cortical excitability (and potential pathophysiology), the effects of stimulus intensity (reflected by different tracking targets) and coil shape on facilitation and inhibition were examined, to thereby determine the role of cortical processes in SICF, SICI, and ICF development. Separately, changes in SICF were correlated with SICI and ICF, to assess whether neuronal disinhibition or potential contamination contributed to the physiological basis of cortical facilitatory and inhibitory phenomena.

## Materials and methods

### Subjects

The study was conducted on consecutively recruited healthy subjects who were without neurological history or psychiatric illness. Hand dominance was determined using the Edinburgh Handiness Inventory (Oldfield, 1971). The study was approved by the Western Area Local Health District ethics committee. Written informed consent was obtained from every subject prior to participation in these experiments.

### Peripheral nerve assessment

Prior to undertaking the assessment of cortical function, peripheral nerve integrity was assessed. Specifically, the median nerve was stimulated electrically at the wrist and the compound muscle action potential (CMAP) was recorded using 10 mm AgCl gel disc electrodes positioned over the dominant hand's abductor pollicis brevis (APB) muscle in a belly tendon montage with a ground electrode placed over the dorsum of the hand. The peak-to-peak CMAP amplitude (mV), onset latency (ms), and F-wave latency (ms) were determined.

### Cortical assessment

#### Study 1: assessing cortical inhibition and facilitation

Subjects were seated comfortably in a purpose-built chair, forearms resting on a pillow midway between pronation and supination. The MEP responses were recorded with the electrodes used in recording CMAP responses, with amplitudes defined by peak-to-peak measurement.

**Figure 1:**
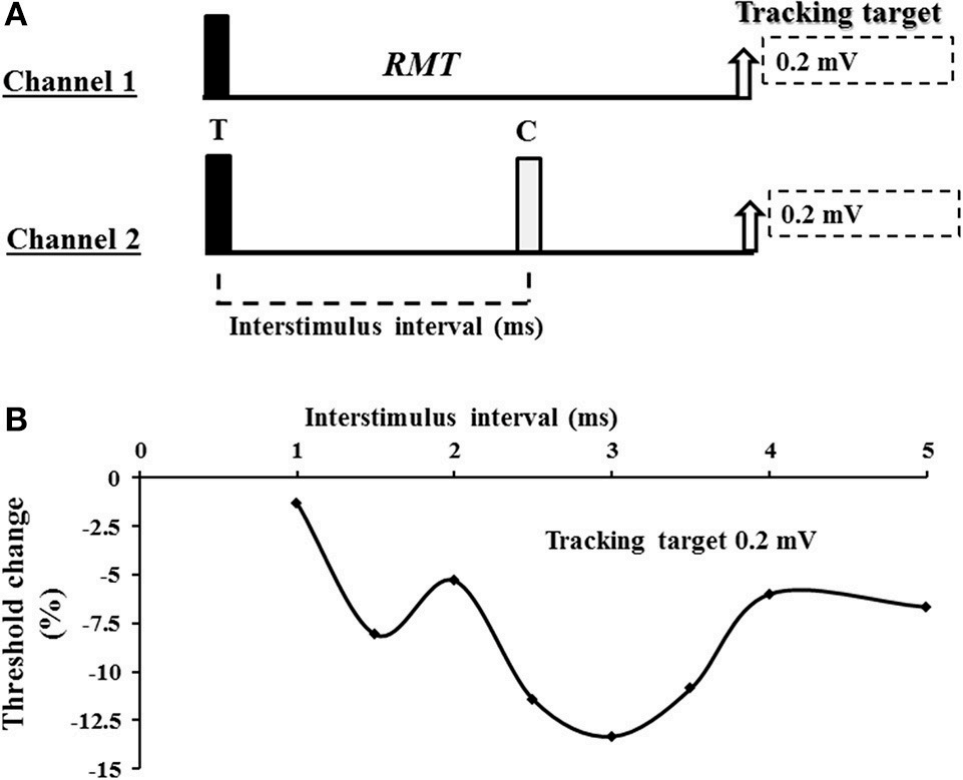
**(A)** Experimental paradigm. Short interval intracortical facilitation (SICF) was recorded using a 2 channel paradigm. On channel 1, an unconditioned stimulus tracked a fixed target of 0.2 or 1.0 mV. The intensity (% maximal stimulator output) required to produce and maintain the tracking target was used to define the resting motor threshold (RMT). On channel 2, the conditioned test response was recorded. For SICF recording, the test response (T) was suprathreshold and preceded the conditioning (C) response which was set to 95% of RMT. **(B)** A SICF recording from a single subject. As described in the Methods and Materials section, SICF was determined by subtracting the stimulus intensity on channel 2 form stimulus intensity recorded by channel 1. In the threshold tracking paradigm, SICF is reflected by negative values indicating that less current is required to produce and maintain the tracking target when compared to the unconditioned (channel 1) stimulus intensity. As such, more negative values indicate a greater degree of SICF.

A 90 mm circular TMS coil, connected to a BiStim^2^ device (Magstim Co., Whitlands, South West Wales, UK), was utilized to generate the MEP responses. The coil position was adjusted to generate an optimal MEP response and subsequently fixed in place using a purpose made stand with additional scalp marking used to corroborate position. The current direction was posterior-anterior in the cortex and the TMS waveform was monophasic. The threshold tracking TMS technique, as previously described (Vucic et al., 2006), was utilized to assess cortical function. Initially, the resting motor threshold was determined and defined as the stimulus intensity required to maintain a target MEP response of 0.2 mV (Vucic et al., 2006). A novel paired pulse threshold tracking paradigm was developed to assess SICF—the conditioning pulse was set to of 95% RMT and positioned after the test pulse (S2 in Figure 1), in keeping with previously reported constant stimulus paradigms (Ziemann et al., 1996c). The intensity of the test pulse varied from stimulus to stimulus as it tracked the target amplitude of 0.2 or 1.0 mV (in study 2). **SICF** was recorded over the following ISIs: 1, 1.5, 2, 2.5, 3, 3.5, 4, 5 ms. Live auditory feedback was maintained throughout the recording session to ensure that the target muscle was at rest. In addition, ***SICI*** was determined over the interstimulus intervals (ISIs) of 1, 1.5, 2, 2.5, 3, 3.5, 4, 5, and 7 ms, while ***ICF*** was assessed over ISIs 10, 15, 20, 25 and 30 ms with conditioning stimulus intensity set to 70% of RMT (Vucic et al., 2006).

SICI, SICF, and ICF were determined using the following formula (Vucic et al., 2006):

$$\begin{array}{l} \text{Inhibition }\left(\text{or Facilitation}\right)\\ =\frac{\text{Conditioned test stimulus intensity}-\text{RMT}}{\text{RMT}}*100 \end{array}$$

#### Single pulse TMS

A resting input-output curve was determined at the following TMS intensities (0.9, 1.0, 1.1, 1.2, 1.3, and 1.5x RMT). Three responses were recorded at each stimulus intensity. The maximum peak-peak maximal MEP amplitude was determined with TMS intensity set to 150% of RMT and expressed as a percentage of the CMAP response. In a subset of the cohort, a facilitated input-output curve (same intensity) was also determined, with subjects maintaining voluntary contraction at 10–20% of maximal effort. The cortical silent period duration was assessed by instructing the subjects were asked to contract the target muscle 20% of maximal voluntary contraction (audio and visual feedback was provided) with the TMS intensities set to 0.9, 1.0, 1.1, 1.2, 1.3, and 1.5x RMT. Three responses were recorded at each intensity and averaged. The CSP duration was measured form onset of facilitation MEP to resumption of EMG activity (Cantello et al., 1992).

The slope of the resting input-output curve was determined using a subset of intensities (0.9, 1.0, 1.1, 1.2, 1.3, 1.5x RMT). TMS intensities were denoted along the horizontal axis (x) and the corresponding average TMS MEP amplitudes along the y-axis. Within this linear model, the least squares method could be utilized to determine slope (b) as follows:

$$\begin{array}{l} b=\frac{\sum \left(x-\bar{x}\right)\left(y-\bar{y}\right)}{\sum {\left(x-\bar{x}\right)}^{2}} \end{array}$$

#### Study 2: determining the effects of target MEP amplitude on intracortical inhibition and facilitation

The experimental procedure with respect to subject position, EMG recording, TMS stimulation timings and conditioner intensities, and digital acquisition of the recording were identical to that of the first study. Initially, the cortical hotspot was re-established according to methodology described in study 1. The RMT, to which the conditioning stimulus was referenced, was defined again as the stimulus intensity required to produce and maintain an MEP response of 0.2 mV (±20%) and determination of RMT was identical to that described in study 1.

The tracking target was varied such that it was set to either an amplitude of 0.2 mV (±20%) or 1.0 mV (±20%). Once tracking of the RMT and 1 mV intensities was reliably determined a paired pulse recording session was undertaken. For recordings with a 0.2 mV tracking target, the QTracS software moved through the three conditions as described earlier. For recordings targeting a 1 mV amplitude, the QTracS software also tracked an additional condition, monitoring the single pulse intensity required to maintain the 1 mV MEP amplitude (1 mV threshold).

SICI, SICF, and ICF for the 1 mV tracking target were determined using the following revised formula:

$$\begin{array}{l} \text{Inhibition }\left(\text{or Facilitation}\right)\\ =\frac{\text{Conditioned test stimulus intensity}-1\text{mV threshold}}{1\text{mV threshold}}*100 \end{array}$$

#### Study 3: assessing the impact of the TMS coil on intracortical inhibition and facilitation

This experimental procedure again proceeded as described in study 1 with respect to matters of subject positioning, EMG recording, and digital acquisition. Cortical excitability was assessed using a circular 90 mm coil and separately the D70 Figure-of-Eight coil (Magstim Co., UK). For both coils, the cortical hotspot for the APB muscle of the dominant hand was established and the RMT determined, with tracking target set to 0.2 mV (±20%). A paired pulse recording capturing SICF, SICI, and ICF was then completed as described in study 1. The choice of which coil that was used first was randomized. For each coil, SICI, SICF, and ICF, together with offline analysis, proceeded as for in study 1.

The MEP and CMAP responses were recorded using a Digitimer D440-4 Amplifier (Digitimer Ltd., UK), bandpass filtered from 10 Hz to 5 KHz. The EMG signal was then digitized using a 16-bit National Instruments (Austin, Texas) Multi-Function IO device (National Instruments USB-6341 DAQ System) sampling at 10 KHz. Data acquisition and stimulation delivery were controlled by QTracS software (Digitimer Ltd., UK).

### Statistical analysis

The Shapiro-Wilks test was used to determined data distribution. For non-parametric data a Friedman's Analysis of Variance by Ranks was used to determine differences. Paired and *post-hoc* testing utilized the Wilcoxon Sign Ranks Test. For parametric data, repeat measures factorial analysis of variance (ANOVA) within the general linear model was used to assess for differences. Paired and *post-hoc* testing utilized the *t*-test. Spearman's Rho or Pearson's correlation coefficients were used for correlations. All data are expressed as mean ± standard error of the mean or median (interquartile range). *P* < 0.05 was deemed statistically significant.

## Results

A total of 35 subjects were recruited (including two left hand dominant and 17 male subjects), with a mean age of 45.6 years (range from 23 to 73 years). None of the subjects withdrew from the study.

### Short interval intracortical facilitation and inhibition

**Figure 2:**
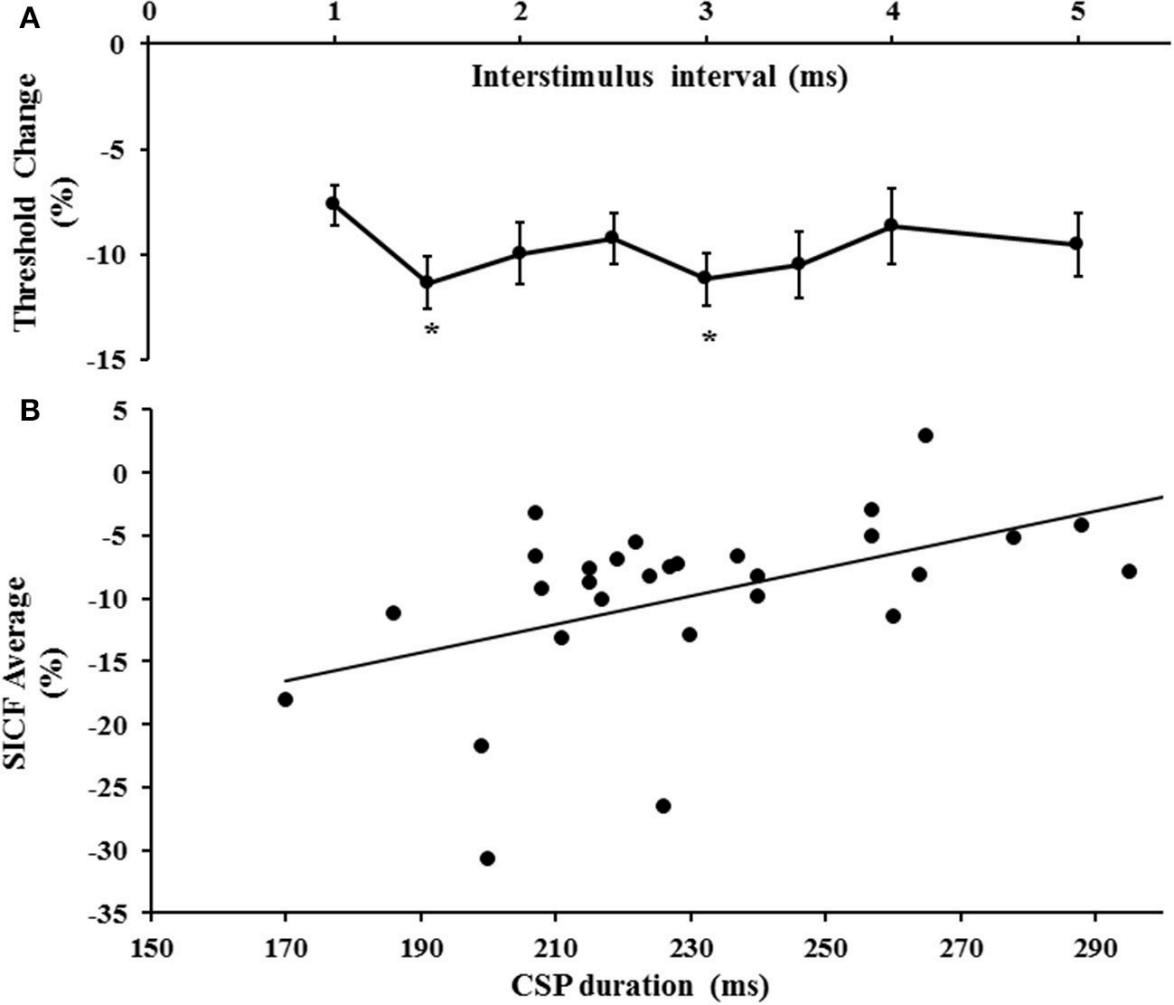
**(A)** Short interval intracortical facilitation (SICF) developed over interstimulus interval (ISI) 1–5 ms. Two peaks (*) at ISI 1.5 and 3 ms were evident. **(B)** There was a significant correlation between SICF and the cortical silent period (CSP) duration.

The first threshold tracking TMS paradigm recorded SICF, which developed between ISIs of 1–5 ms (Figure 2A). Importantly, the threshold changes evident for SICF at each ISI were significantly different from the resting motor threshold (*X*^2^ of 51.610, *P* < 0.001), a notion confirmed on *post-hoc* testing (*P* < 0.001), and there was a significant correlation of SICF recorded at each of the ISI levels (Table 1). Two distinct SICF peaks were evident at ISIs 1.5 ms (−11.35 ± 1.23%) and 3 ms (−11.19 ± 1.26%, Figure 2A), with the averaged SICF between ISIs 1–5 ms being −9.77 ± 1.12%, although facilitation was evident across all ISIs tested.

**Table 1:** Correlation of short interval intracortical inhibition at each interstimulus interval (ISI).

**ISI (ms)**	**1**	**1.5**	**2**	**2.5**	**3**	**3.5**	**4**	**5**
1		0.836^**^	0.586^**^	0.468^**^	0.398^*^	0.355^*^	0.349^*^	0.184
1.5	0.836^**^		0.719^**^	0.485^**^	0.527^**^	0.503^**^	0.451^**^	0.340^*^
2	0.586^**^	0.719^**^		0.665^**^	0.615^**^	0.676^**^	0.665^**^	0.454^**^
2.5	0.468^**^	0.485^**^	0.665^**^		0.832^**^	0.714^**^	0.758^**^	0.527^**^
3	0.398^**^	0.527^**^	0.615^**^	0.832^**^		0.852^**^	0.796^**^	0.551^**^
4	0.349^**^	0.451^**^	0.665^**^	0.758^**^	0.796^**^	0.932^**^		0.733^*^
5	0.184	0.340^*^	0.454^**^	0.527^**^	0.551^**^	0.682^**^	0.733^**^	

*The R-values are expressed*.

* P < 0.05;

** *P < 0.01*.

**Figure 3:**
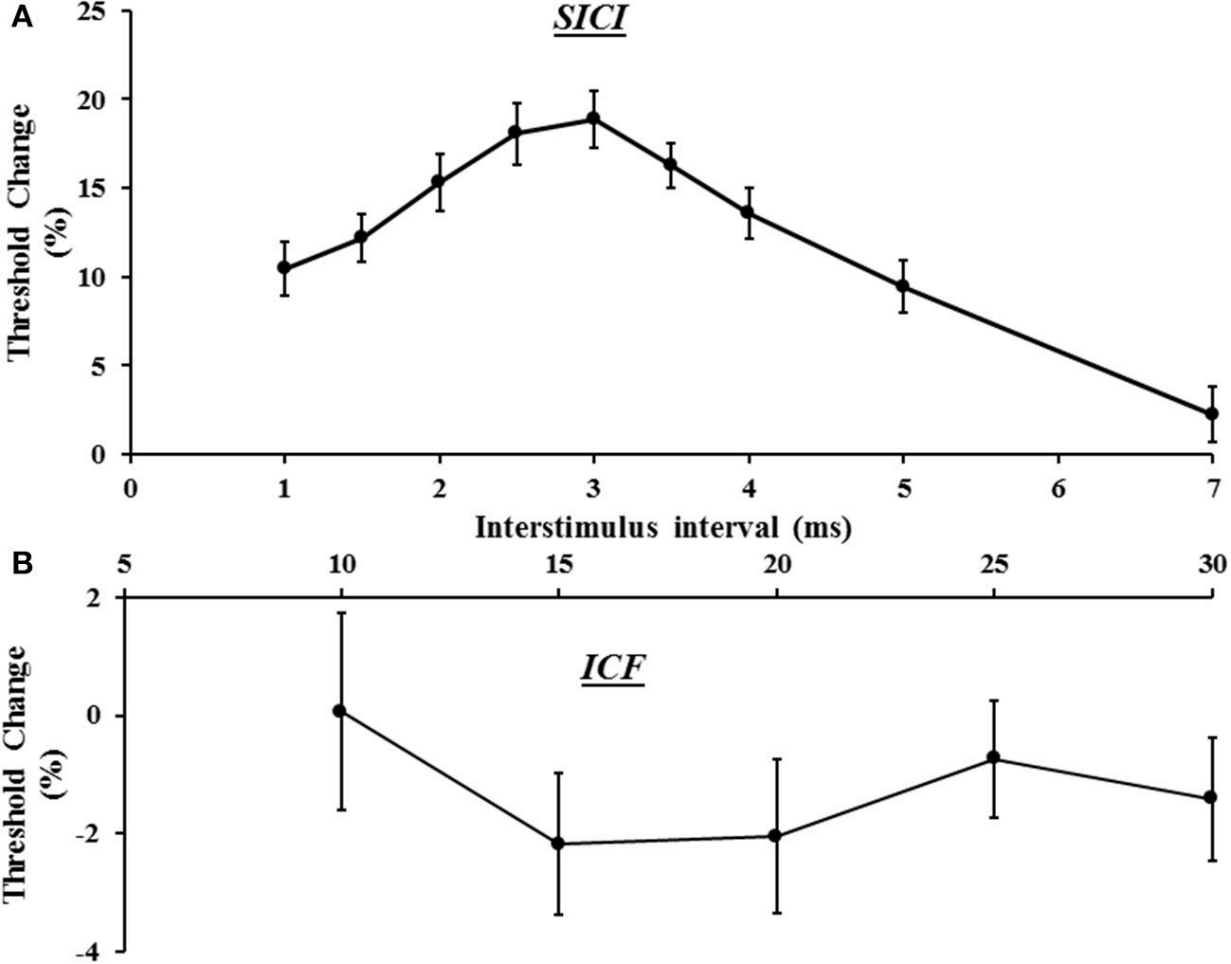
**(A)** Short interval intracortical inhibition (SICI) developed between interstimulus intervals (ISI) 1–7 ms, with peak SICI occurring at ISI 3 ms. **(B)** Intracortical facilitation (ICF) was evident between ISIs 10–30 ms, peaking at ISI 15 ms.

In the same sitting, SICI was assessed between ISIs 1–7 ms, peaking at ISI 3 ms (Figure 3A), with mean SICI between ISI 1–7 ms being 18.88 ± 1.61% and in keeping with previous findings (Vucic et al., 2006). As previously reported, ICF developed between ISIs 10–30 ms, peaking at ISI 15 ms (Figure 3B). Repeated measures ANOVA, with a single factor (paired pulse paradigm) and three levels (the average SICF, SICI, and ICF), disclosed a significant difference between the three TMS parameters (*F* = 159.25, *P* < 0.001).

Correlation studies disclosed a significant correlation between averaged SICF and cortical silent period duration (*r* = 0.518, *P* = 0.003, Figure 2B). There was no significant correlation between mean SICF and mean SICI (*P* = 0.735), peak SICI at ISI 3 ms (*P* = 0.521), ICF (*P* = 0.302), maximal MEP amplitude (Rest *P* = 0.452, Active *P* = 0.675), RMT (*P* = 0.657), or the input/output curves (Rest *P* = 0.291, Active *P* = 0.097).

### The effect of target amplitude

**Figure 4:**
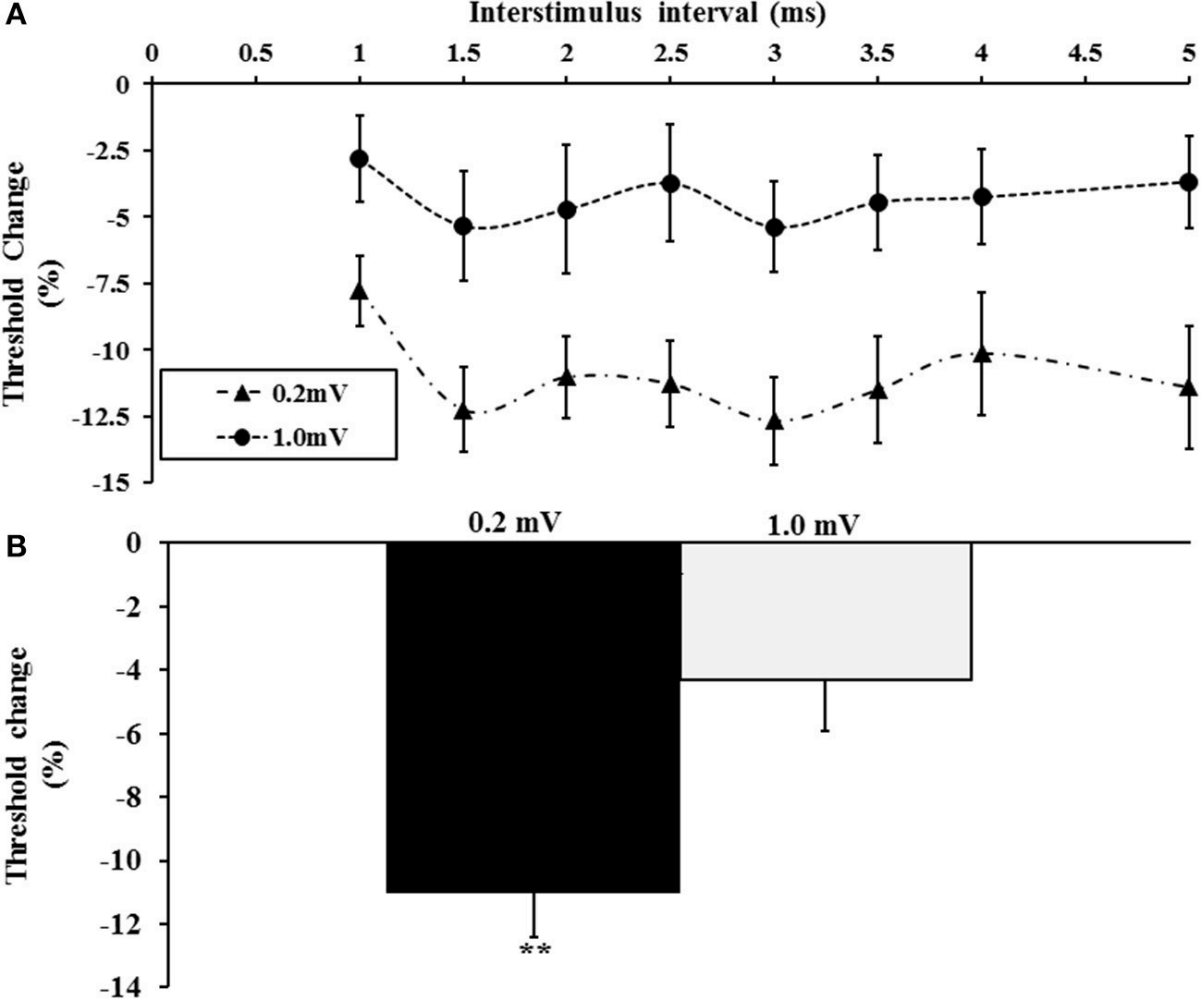
**(A)** Short interval intracortical facilitation (SICF) was significantly increased when tracking a target of 0.2 mV. **(B)** The averaged SICF, between interstimulus interval 1–5 ms was significantly increased with the 0.2 mV tracking target. **P < 0.01.

Here the effects of the tracking target amplitude (0.2 and 1 mV) on intracortical facilitation and inhibition were assessed. A total of 20 subjects (mean age 41 years, range 26–65 years, 18 right handed, 10 males) were assessed in this experiment with the tracking target set to 0.2 or 1 mV. The single pulse threshold was significantly higher with the tracking target set to 1.0 mV (63.6 ± 2.5%) when compared to 0.2 mV (51.9 ± 1.7%). A two-factor repeated measure ANOVA using target intensity (0.2 vs. 1.0 mV) and interstimulus intervals (ISI 1–5 ms) as factors, disclosed a significant effect of target level on SICF (*F* = 5.631, *P* < 0.05) with the SICF being significantly reduced when the tracking target was set to 1 mV (Figure 4A). Interestingly, there was no significant effect of ISI (*F* = 2.318, *P* = 0.09) or ISI^*^target level (*F* = 0.504, *P* = 0.642) on SICF. The averaged SICF (ISI 1–5 ms) was significantly greater with the tracking target set to 0.2 mV (−11.0 ± 1.4%) when compared to a tracking target of 1.0 mV (−4.3 ± 1.6%, *P* < 0.01, Figure 4B).

**Figure 5:**
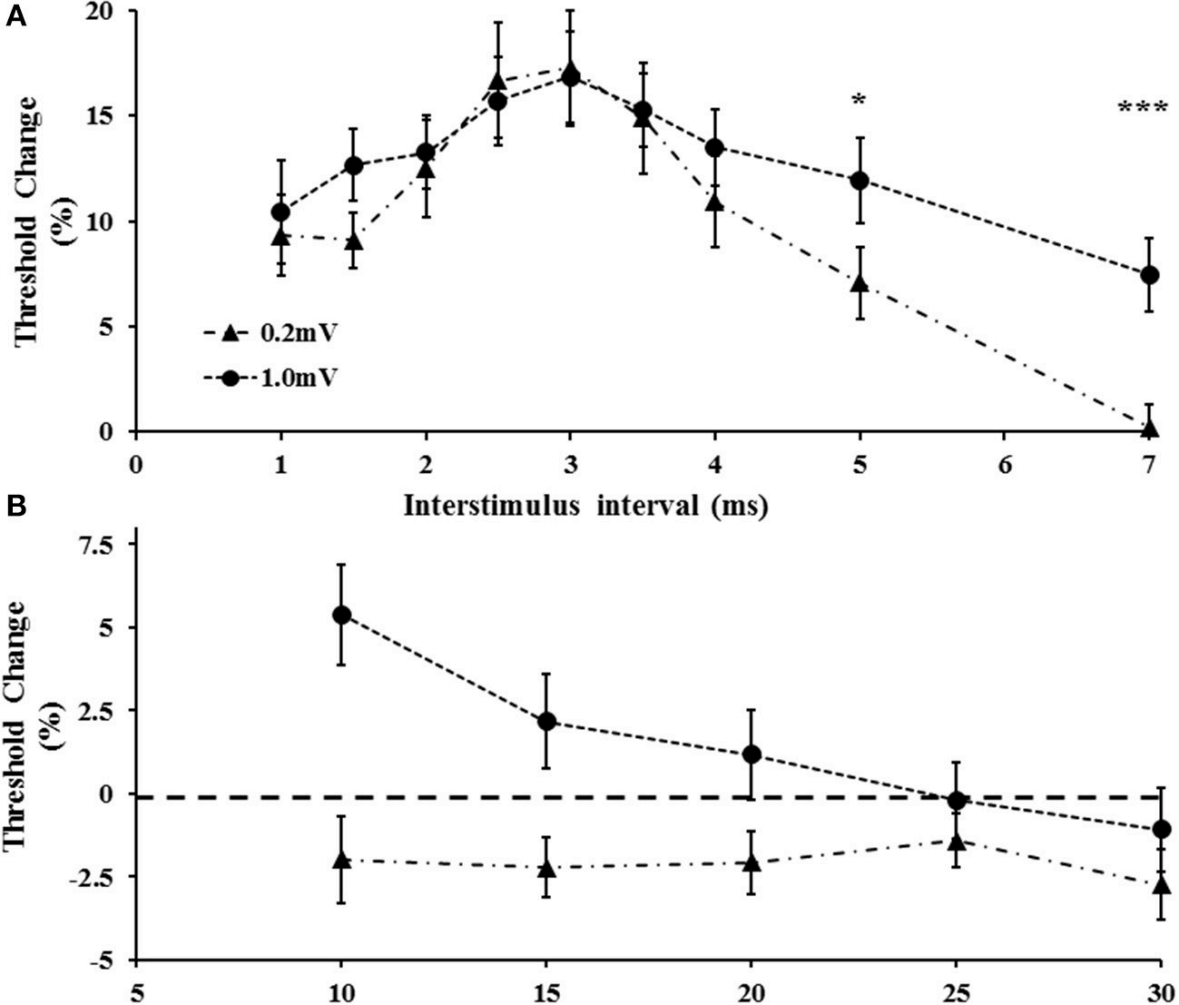
**(A)** With tracking target set to 1.0 m V, short interval intracortical inhibition was increased. The increase was evident at interstimulus intervals of 5 and 7 ms. **(B)** Intracortical facilitation was significantly reduced at the higher tracking target (1.0 mV). *P < 0.05; ***P < 0.001.

Separately, a two-factor repeated measure ANOVA using target intensity (0.2 vs. 1.0 mV) and interstimulus intervals (ISI 1–7 ms) as factors, disclosed that the target level (*F* = 5.124, *P* < 0.05) and ISI (*F* = 11.240, *P* < 0.001) exerted independent effects on SICI, but that the target level^*^ISI (1.667, *P* = 0.112) did not independently influence SICI. Specifically, there was a significant increase of SICI at ISI 5 and 7 ms when the target amplitude was set to 1.0 mV (Figure 5A). Of further relevance, the target level also exerted significant effects in ICF (Figure 5B). Specifically, a two factor ANOVA disclosed a significant impact of target (*F* = 12.089, *P* < 0.01) and ISI (*F* = 3.39, *P* < 0.05) in isolation, as well as target intensity^*^ ISI (*F* = 6.331, *P* < 0.01) on ICF. As illustrated in Figure 5B, ICF was not identified at ISIs 10, 15, 20 ms (*P* < 0.05) when the target level was set to 1.0 mV.

Correlation studies were undertaken to assess whether there were any associations between SICF, SICI, and ICF at different tracking targets. There was a significant inverse correlation between SICF at 0.2 mV and SICF at 1 mV (*R* = −0.516, *P* < 0.05). In addition, there was a positive correlation between SICI (*R* = 0.417, *P* < 0.05) measured at the two tracking target levels, a finding also demonstrated for ICF (*R* = 0.385, *P* < 0.05). Importantly, there was no correlation between SICF and SICI or ICF at and across both target amplitude levels (all *P* > 0.05), suggesting that underlying potential strength of SICF for individual subjects did not in fact influence SICI.

### The influence of age on paired pulse paradigms

To examine for the potential effect of age on interneuronal facilitatory and inhibitory activity we performed two analyses. Firstly we performed a correlation analyses for each of SICI, SICF, and ICF. A significant correlation with age was not found for SICF (*r* = 0.037, *p* = 0.835), SICI (*r* = 0.183, *p* = 0.293), or ICF (*r* = 0.288, *p* = 0.099). We next divided our cohort into two groups based on age including 18 younger subjects (mean 33.89, range 23–43), and 17 older subjects (mean 57.94, range 44–73). Using an independent samples *t*-test there were no significant differences between older and younger subjects for SICF (Younger = −9.42 ± 1.30, Older = −10.12 ± 1.88 *p* = 0.757), SICI (Younger = 13.18 ± 1.51, Older = 13.38 ± 1.37 *p* = 0.924), and ICF (Younger = −0.89 ± 1.29, Older = −0.48 ± 1.05, *p* = 0.806).

### Circular vs. figure-of-eight coils

**Figure 6:**
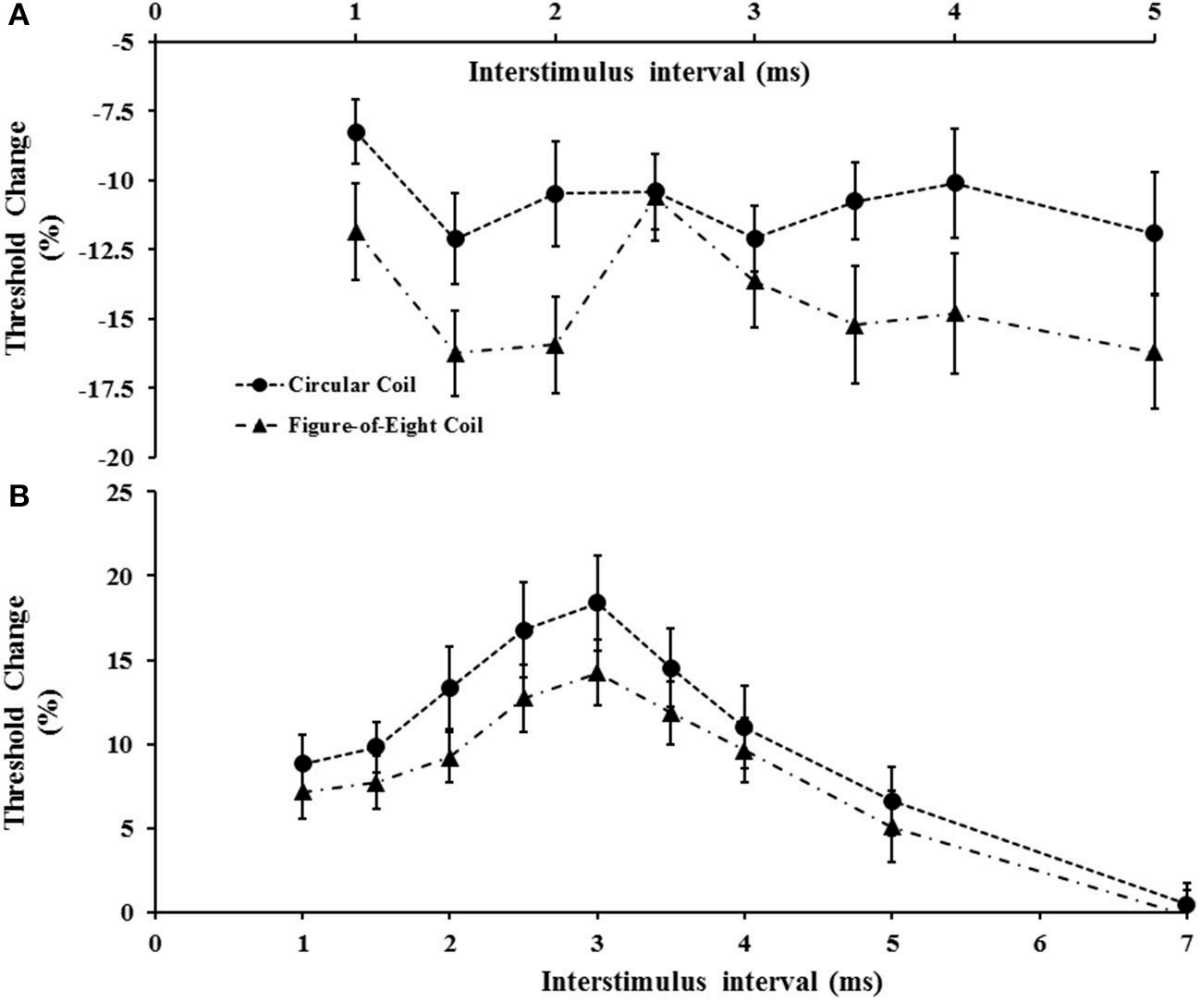
**(A)** Short interval intracortical facilitation (SICF) was significantly increased with the figure-of-eight coil. At interstimulus interval (ISI) 2.5 ms there was a significant reduction of SICF compared to neighboring ISI values (2 and 3 ms) when utilizing the figure-of-eight coil. **(B)** Short interval intracortical inhibition was significantly increased when using the circular coil.

The impact of coil shape on intracortical facilitation and inhibition was also assessed by examining the effects of the circular and figure-of-eight shaped coils. The figure of eight coil resulted in a significant increase in SICF (Figure 6A). Specifically, the two factor repeated measures ANOVA (factors of ISI and coil type) disclosed a significant impact of coil type (*F* = 6.208, *P* < 0.05) on SICF, but no significant effect of ISI (*F* = 2.672, *P* = 0.055) or ISI^*^coil type interaction (*F* = 1.505, *P* = 0.227). The averaged SICF between ISI 1–5 ms, was significantly larger using figure of eight-coil (−14.3 ±1.4%) compared to the circular coil (−10.8 ± 1.2%, *P* < 0.05).

Separately, the coil shape (*F* = 15.911, *P* < 0.001), and ISI (*F* = 4.428, *P* < 0.05), exerted independent effects on SICI but not a combination of coil type^*^ISI (*F* = 0.883, *P* = 0.522). The mean SICI (figure-eight, 8.6 ± 1.2%; circular coil, 11.1 ± 1.7%, *P* < 0.05) and peak SICI at 3 ms (*P* < 0.05, Figure 6B) were significantly reduced when stimulating with the figure of eight coil. In contrast, coil shape did not exert any significant effect on ICF, with the mean ICF elicited by the figure of eight coil (−3.1 ± 1.2%) comparable to that produced by the circular coil (−2.0 ± 1.2%, *P* = 0.435).

There was a significant correlation between SICF generated by the figure of eight and circular coils (*R* = 0.40, *P* < 0.05), as well as SICI generated by the two coils (*R* = 0.684, *P* < 0.001). When using the figure of eight coil, there was again no significant correlation between SICF and SICI.

## Discussion

Utilizing the paired-pulse threshold tracking TMS technique, the present study interrogated the facilitatory and inhibitory processes contributing to cortical excitability in 35 healthy human controls. SICF developed between interstimulus intervals of 1–5 ms, peaking at 1.5 and 3 ms, a finding consistent with the I-wave periodicity. Importantly, both the tracking target level and coil shape influenced SICF, such that SICF was higher with the lower tracking target (0.2 mV) and the figure-of-eight coil. Although SICF developed over a similar time course as SICI (ISI 1–7 ms), the two phenomena appeared to be independent with no correlation between paradigms. Furthermore strengthening and weakening of SICF or SICI with parameters such as target amplitude and coil type were not transferred across paradigms. In addition, ICF and SICF appeared to reflect distinct facilitatory processes, developing over different time course and again differentially influenced by tracking target levels and coil shape. A separate analysis of SICF, SICI, and ICF found no significant influence of age within our cohort of healthy adults.

While the physiological mechanisms underlying the development of facilitatory and inhibitory phenomenon appear complex, the role of cortical circuits in contributing to these processes will be discussed.

### Cortical processes mediating SICF

Prior to undertaking a discussion on the physiological mechanisms underlying the development of SICF, it would seem prudent to discuss whether the SICF recorded with different TMS methodologies reflected a similar physiological process. Using a similar stimulation paradigm, but a constant stimulus method, previous studies have reported that SICF developed over an interstimulus interval range of 1–5 ms (Tokimura et al., 1996; Ziemann et al., 1998a). In addition, three distinct SICF peaks were identified, occurring at ISIs 1–1.5, 2.5–3, and around 4.5 ms, which were accompanied by troughs. In the present study, SICF developed over a similar time course (ISI 1–5 ms) with two distinct peaks occurring at ISI 1.5 and 3 ms, although the previously reported periodicity was less distinct. It seems likely that the SICF recorded using the threshold tracking technique reflects a similar physiological process as that measured by the constant stimulus method. The discordant findings could relate to differences in the TMS methodology, whereby the larger peaks and troughs detected with the constant stimulus method perhaps reflecting the larger variability in the outcome variable, namely the MEP amplitude (Kiers et al., 1993). The threshold tracking strategy “targets” a fixed MEP amplitude that lies in the steepest portion of the input/output curve (Fisher et al., 2002; Vucic et al., 2006), thereby obviating the large variation in the outcome variable.

Although the mechanisms underlying SICF development remain to be fully elucidated, it has been proposed that facilitatory interactions of I-waves at a motor cortical level form the basis of SICF (Ziemann et al., 1998a, 2015). Modeling studies of TMS induced I-waves have suggested that the suprathreshold S1 stimulus leads to a variable and incomplete activation of motor cortical neurons (Rusu et al., 2014), resulting in subliminal depolarization of a subpopulation of cortical neurons. A subsequent subthreshold stimulus (S2) applied at short ISIs, causes the subliminally depolarized neurons to reach threshold, thereby generating an MEP potential and facilitation. Support for a cortical origin of SICF was suggested by the observed periodicity of SICF peaks, which occur at 1.5 ms (~660 Hz), and are consistent with the I-wave frequency (Amassian et al., 1987). This notion was further supported by pharmaco-TMS studies documenting a modulating effect on SICF by a variety of neurotransmitter systems (Ilić et al., 2002, 2003; Korchounov and Ziemann, 2011; Ziemann et al., 2015), all of which control the neuronal circuitry underlying I-wave generation (Di Lazzaro and Ziemann, 2013). Specifically, previous studies has reported a reduction of SICF with GABA and dopamine agonists, such as lorezapam and cabergoline respectively, while Na^+^ agonists, GABA_B_ antagonists and NMDA receptor antagonists do not modulate SICF (Inghilleri et al., 1996; Ziemann et al., 1996a,b, 1998b, 2015; Boroojerdi et al., 2001; Ilić et al., 2002). In addition, the facilitating effects of SICI on SICF (Wagle-Shukla et al., 2009), provided additional evidence for the importance of cortical neuronal circuitry in SICF development through disynaptic inhibition of I-wave generating interneurons. The notion of disynaptic inhibition, however, was not a uniform finding, with one study suggesting that SICF contaminated the process of SICI (Peurala et al., 2008).

The findings in the present study would support the hypothesis that I-wave facilitation underlies the development of SICF. Specifically, the stimulation paradigm implemented in the recording of SICF (supra-threshold S1 followed by subthreshold S2) along with the presence of two SICF peaks (at ISI 1.5 and 3 ms), which resemble the periodicity of I-waves, would support the I-wave hypothesis. Of further relevance, the significant correlation of SICF across different ISIs (Table 1), suggests that similar mechanisms were mediating SICF at specific ISIs, most likely at a cortical level. The notion of disynaptic inhibition and SICF contamination of SICI, however, was not substantiated in the present study as there was no correlation between mean SICF and SICI, and the two curves were significantly different over the interstimulus interval of 1–7 ms. A potential explanation for the discordant findings between the present study and previous studies invoking disynaptic inhibition (Wagle-Shukla et al., 2009) and SICF/SICI interaction (Peurala et al., 2008) may relate differences in subject numbers and the methodologies used to record SICI. Alternatively, variations in the anatomical arrangements of interneuronal circuits underlying SICF and SICI (Hamada et al., 2013), could also account for these discordant findings.

Surprisingly there was a significant correlation between the CSP duration and SICF in the current cohort, whereby longer CSP durations were associated with reduced SICF. It is well-established that CSP duration is in part mediated by long-lasting cortical inhibitory circuits acting via GABA_B_ receptors (Connors et al., 1988; Nakamura et al., 1997; Werhahn et al., 1999; Ziemann et al., 2015). While the precise mechanism underlying this association remains obscure, it could be argued that reduction in the activity of long lasting cortical inhibitory circuits acting via GABA_B_ receptors contributed to the development of SICF. Given that spinal inhibition contributes to the early component of the CSP duration (Inghilleri et al., 1993), assessing the effects of long interval intracortical inhibition (mediated through GABA_B_ receptors; Werhahn et al., 1999) on SICF, could further clarify the importance of GABA_B_ receptor mediated disinhibition in SICF.

#### Effects of increasing TMS intensity

Increasing the tracking target amplitude from 0.2 to 1.0 mV resulted in a significant reduction of SICF, which is in keeping with a previous study (Wagle-Shukla et al., 2009). While the physiological processes mediating SICF reduction with higher TMS intensities remain to be fully clarified, it could relate to refractoriness of the cortical neuronal pool. Specifically, higher S1 intensities would activate a greater population of cortical neurons, thereby depleting the pool of subliminally depolarized neurons which would be activated by the subthreshold (S2) stimulus. In addition, the higher TMS intensities required to generate MEP amplitudes of 1.0 mV could lead to greater recruitment of later I-wave activity and near maximal recruitment of early I-wave activity, thereby resulting in more SICI and less SICF. Alternatively, it could be argued that higher TMS intensities stimulated inhibitory circuits, which are located in the deeper layers of the motor cortex or are distant to the M1 cortex (DeFelipe et al., 2013), thereby resulting in an inhibitory contamination and reduction of SICF. While the latter possibility was not completely excluded, the significant correlation between SICF at the two tracking levels, preservation of the two SICF peaks and identical time course of SICF development, would suggest that similar physiological processes were operating at both tracking levels, albeit to a lesser degree with the tracking target at 1.0 mV. As such, depletion of the subliminally depolarized corticomotoneuronal pool could account for the lesser degree of facilitation evident at the higher tracking target.

In addition, intracortical facilitation was absent while SICI increased with the 1.0 mV tracking target, a finding consistent with previous studies (Sanger et al., 2001; Daskalakis et al., 2002; Wagle-Shukla et al., 2009). It is well-established that SICI and ICF are mediated by different cortical networks (Ziemann et al., 1996c). Increases in SICI evident with higher conditioning (S1) intensities, and mimicked by setting the tracking target to 1.0 mV, may be explained by recruitment of later I-waves which contribute to SICI development (Di Lazzaro et al., 1998b; Hanajima et al., 2003). The mechanisms governing the reduction in ICF with higher stimulus intensities appears to be complex and related to recruitment of corticomotoneuronal circuits that are less sensitive to facilitation, are more distant from the TMS stimulation site (Volz et al., 2015), or involve recruitment of spinal processes (Di Lazzaro et al., 2006). In addition, an inhibitory contribution of the SICI tail (Hanajima et al., 1998) could also account for ICF reduction evident with higher stimulus intensities, a notion supported in the present study by findings of significant correlation between the SICI tail (at ISI 5 and 7 ms) and the early segments of ICF (at 10 ms).

Of further interest was the significant correlation between SICI at 1.0 mV and cortical silent period duration, a finding not evident when SICI was tracked at 0.2 mV. As discussed above, the CSP duration appears to be mediated by a combination of spinal inhibitory circuits and cortical inhibitory processes acting via GABA_B_ receptors (Connors et al., 1988; Nakamura et al., 1997; Werhahn et al., 1999; Ziemann et al., 2015). Higher TMS intensities could activate these spinal and cortical inhibitory processes, which could function in concert with the inhibitory GABA_A_ acting circuits, and contribute to SICI. Consequently, future studies utilizing the threshold tracking TMS technique to assess SICI changes in neurological diseases should utilize a 0.2 mV tracking target in order to avoid contamination from non-GABA_A_ acting inhibitory circuits.

#### Effects of coil shape

In the present study, the shape of the TMS coil significantly impacted on SICF. Specifically, stimulation with a figure of eight coil significantly increased SICF and altered the shape of the SICF curve such that peak SICF at ISI 3 ms was no longer prominent and there appeared to be a relative reduction of SICF at ISI 2.5 ms. Importantly, there was a significant correlation between SICF values recorded with the different coils, implying similar underlying physiological processes. The figure-of-eight coil induces a more focal cortical stimulation, and when positioned to induce currents in the posterior-to-anterior direction, as in the present study, leads to a preferential recruitment of early I-waves at low-moderate TMS intensities (Day et al., 1987, 1989; Werhahn et al., 1994; Di Lazzaro et al., 2001). Consequently, more effective stimulation of the high threshold cortical neuronal circuits, which underlies I-wave recruitment and thereby SICF, could account for the present findings. Separately, circular coil stimulation has been shown to evoke D waves originating at the level of the initial segment of the axon is evoked, whilst with the figure-of eight coil (P-A) D waves can be generated with higher intensities at some distance from the cell body (Di Lazzaro et al., 2002, 2017). Consequently, antidromic propagation of the proximal D-wave with circular coil stimulation may render some corticospinal cells refractory to I1 wave inputs, thereby resulting in reduced SICF.

In contrast, circular coil induces a larger magnetic field, leading to a greater area of cortical stimulation and recruitment of a mixture of D and I-waves (Burke et al., 1993), including the recruitment of later I-waves (I3) which are associated with SICI development (Di Lazzaro et al., 1998b). Consequently, the circular coil may be more effective at stimulating the cortical inhibitory processes, thereby preferentially increasing SICI, which was evident in the present study. Alternatively it could be argued that in advertent activation of transcallosal inhibitory fibers by the non-selective stimulation with the circular could account for the increase in SICI. This seems unlikely given the short latencies used for SICI (1–7 ms) and the fact previous studies have reported a reduction of SICI with short latency IHI (Daskalakis et al., 2002; Florian et al., 2008).

In conclusion, findings in the present study establish SICF as a novel threshold tracking TMS parameter and suggest that SICF is distinct from both SICI and intracortical facilitation. The periodicity of the SICF peaks along with the short latency time course (developing of ISI 1–5 ms) imply a cortical origin of SICF, a notion supported by observation of SICF reduction with higher stimulus intensities (tracking target 1 mV) and increases with the use of a more specific TMS coil. The lack of correlation between SICF and SICI argues against disynaptic disinhibition as a significant mechanism in the development of SICF. Additionally, the finding of a significant correlation between SICF and CSP duration suggests a contribution of long latency inhibitory cortical processes, perhaps via GABAB_B_ receptor mediated presynaptic disinhibition which is distinct from those mediating SICI. Future studies should assess the utility of SICF as a pathophysiological biomarker in neurodegenerative diseases, such as amyotrophic lateral sclerosis.

## Author contributions

MB: collected all data, performed analysis, and wrote manuscript; PM: collected data and edited manuscript; NG: analyzed data and edited manuscript; JH: edited manuscript; MK: edited manuscript and provided critical insights; SV: conceived the study, analyzed data, edited manuscript.

### Conflict of interest statement

The authors declare that the research was conducted in the absence of any commercial or financial relationships that could be construed as a potential conflict of interest.
